# 12-Month Outcome Data for Buprenorphine-Naloxone Maintenance Treatment in Individuals with Opioid Use Disorder

**DOI:** 10.1192/j.eurpsy.2024.527

**Published:** 2024-08-27

**Authors:** Y. Taylan, M. B. Sönmez

**Affiliations:** ^1^İstanbul Sultanbeyli State Hospital, İstanbul; ^2^Trakya University School of Medicine, Edirne, Türkiye

## Abstract

**Introduction:**

Buprenorphine/Naloxone (B/N) is a safe and effective treatment for the long-term stabilization of individuals with opioid use disorder (OUD). Patients undergoing opioid maintenance treatment experience reduced mortality rates, decreased substance use, and an overall improvement in their quality of life. Premature discontinuation of maintenance treatment increases the risk of relapse.

**Objectives:**

Our primary objective was to assess patient compliance with maintenance treatment and to identify potential factors associated with treatment discontinuation and relapse.

**Methods:**

The study involved 206 patients with OUD who initially enrolled in a 28-day abstinence-based inpatient program at our hospital. Following their inpatient treatment, they were subsequently admitted as outpatients for B/N maintenance treatment at the Alcohol and Substance Addiction Treatment Center in Trakya University School of Medicine (Edirne, Türkiye). The addiction profiles of patients were assessed using the Addiction Profile Index (API) Clinical Form during the baseline evaluation. Sociodemographic and clinical data were collected from the patients’ records.

**Results:**

After 3 months, 114 patients (55.3%) remained in treatment, and 52 patients (25.2%) were still in treatment at the end of 1 year. Factors associated with a higher likelihood of remaining in treatment for one year included older age (z=-2.257, p=0.024), longer length of education (z=-2.270, p=0.023), later onset of smoking (z=-2.704, p=0.007), later onset of substance use (z=-3.597, p<0.001), and a higher rate of completing the inpatient treatment program (χ²=4.016, p=0.045). Patients in the 1-year retention group had lower scores on the API anxiety (z=2.767, p=0.009), anger management problems (z=2.754, p=0.011), and novelty-seeking behavior (z=2.634, p=0.043) subscales. They also had a lower rate of having a criminal history (χ²=5.349, p=0.021). The duration of treatment retention was positively correlated with age (r=0.160, p=0.021), length of education (r=0.158, p=0.023), age of onset of smoking (r=0.228, p=0.001), and age of onset of substance use (r=0.268, p<0.001). It was negatively correlated with the duration of substance use (r=-0.138, p=0.048), the number of inpatient treatments (r=-0.142, p=0.042), and scores on the API anxiety (r=-0.167, p=0.040), anger management problems (r=-0.173, p=0.033), and novelty-seeking behavior (r=-0.209, p=0.010) subscales.

**Conclusions:**

Identifying the specific factors associated with treatment retention and dropout/relapse can be valuable in developing more effective and personalized treatment plans for individuals with OUD.

**Disclosure of Interest:**

None Declared

